# Nature-inspired metaheuristics for optimizing dose-finding and computationally challenging clinical trial designs

**DOI:** 10.1177/17407745251346396

**Published:** 2025-07-12

**Authors:** Weng Kee Wong, Yevgen Ryeznik, Oleksandr Sverdlov, Ping-Yang Chen, Xinying Fang, Ray-Bing Chen, Shouhao Zhou, J Jack Lee

**Affiliations:** 1 Department of Biostatistics, University of California, Los Angeles, CA, USA; 2 Department of Pharmacy, Uppsala University, Uppsala, Sweden; 3 Advanced Quantitative Sciences, Novartis Pharmaceuticals Corporation, East Hanover, NJ, USA; 4 Department of Statistics, National Taipei University, New Taipei City; 5 Department of Public Health Sciences, Pennsylvania State University, Hershey, PA, USA; 6 Institute of Statistics and Data Science, National Tsing Hua University, Hsinchu; 7 Department of Biostatistics, The University of Texas MD Anderson Cancer Center, Houston, TX, USA

**Keywords:** Continuation-ratio model, dose-finding trial, optimal biological dose, particle swarm optimization, phase I/II trial

## Abstract

Metaheuristics are commonly used in computer science and engineering to solve optimization problems, but their potential applications in clinical trial design have remained largely unexplored. This article provides a brief overview of metaheuristics and reviews their limited use in clinical trial settings. We focus on nature-inspired metaheuristics and apply one of its exemplary algorithms, the particle swarm optimization (PSO) algorithm, to find phase I/II designs that jointly consider toxicity and efficacy. As a specific application, we demonstrate the utility of PSO in designing optimal dose-finding studies to estimate the optimal biological dose (OBD) for a continuation-ratio model with four parameters under multiple constraints. Our design improves existing designs by protecting patients from receiving doses higher than the unknown maximum tolerated dose and ensuring that the OBD is estimated with high accuracy. In addition, we show the effectiveness of metaheuristics in addressing more computationally challenging design problems by extending Simon’s phase II designs to more than two stages and finding more flexible Bayesian optimal phase II designs with enhanced power.

## Introduction

Nature-inspired metaheuristic algorithms have been widely used in computer science and engineering to tackle complex optimization problems for at least the last three decades.^1–4^ Their popularity has skyrocketed in both industry and academia, spreading across various disciplines.^5–7^ These algorithms, inspired by natural phenomena such as animal behavior, are employed in diverse research areas, including machine learning.^8,9^ Each algorithm begins with a randomly generated set of candidate solutions, known as particles, and the number of particles used in the search is referred to as the swarm size. These algorithms incorporate stochastic components and tuning parameters, with default settings typically performing well. During each iteration, the particles improve their proximity to the global optimum, with each algorithm employing different methods for their improvement. Generally, these algorithms are fast, easy to implement, and often capable of finding a solution or an approximate solution to the optimization problem. They are intriguing because they do not require technical assumptions to work effectively, despite lacking rigorous proof of convergence. Consequently, these algorithms are sometimes referred to as general-purpose optimization tools or last-resort algorithms, meaning they should be used when other optimization methods fail.

In recent years, there has been a noticeable increase in papers using nature-inspired metaheuristic algorithms to address challenging optimal design problems in the statistics literature. The trend is primarily due to the limitations of traditional optimal design models, which often involve a few variables and assume additivity for analytical derivations. As models become more complex, these assumptions become impractical. While numerical approaches are useful, many are ad hoc and limited in scope. They tend to perform well for low-dimensional problems but struggle with high-dimensional optimization problems, even when the algorithm has proof of convergence. Metaheuristics have shown potential in overcoming these computational challenges. Recent publications have demonstrated their utility and flexibility in finding optimal designs for nonlinear models with multiple interacting factors.

An exemplary nature-inspired algorithm is particle swarm optimization (PSO). It is highly popular, and numerous modifications, known as variants, have been developed to enhance its performance in various ways. Qiu et al.^10^ and Lukemire et al.^11,12^ have utilized these variants to address diverse optimal design problems for various statistical nonlinear models. These include high-dimensional optimal design problems with multiple interacting variables, as well as problems with non-differentiable or implicitly defined objective functions. One example of a design problem with a non-differentiable criterion is the standardized maximin criterion, where the goal is to find a design that maximizes the minimal D-inefficiency across all designs, with unknown parameters assumed to belong to a user-selected plausible region.^13^ Another example involves design problems with implicitly defined objective functions, such as the case studied here. Lukemire et al.^11,12^ also applied metaheuristics to obtain optimal designs for various statistical models, including Bayesian optimal designs. Their flexibility extends to finding optimal designs for quantile regression models.^14^

The aim of this article is to introduce nature-inspired metaheuristics to researchers in clinical trials and demonstrate their usefulness in finding flexible and practical dose-finding designs. There are many such algorithms, including genetic algorithms (GAs), differential evolution (DE), PSO, and various PSO variants. These algorithms share common features: they begin by randomly generating a user-specified pool of candidate solutions (particles) to search for a global optimum, and they explore and exploit the search domain in different ways. The algorithm stops when it reaches the specified number of function evaluations or iterations, or when it finds the optimal solution based on a pre-specified tolerance level. Metaheuristic algorithms have several commonalities, including (a) stochastic components; (b) tuning parameters; (c) variants; and (d) hybridization.

*Stochastic components* introduce randomness into the search process, allowing the algorithm to escape from local optima. Each algorithm typically has a few *tuning parameters* that define the behavior of the search agents. Algorithm proponents often provide default values for these tuning parameters, and the algorithm’s performance can depend significantly on these defaults. *Variants* are modifications of the original metaheuristic algorithms designed to enhance their performance. For example, a variant might achieve faster convergence, be less sensitive to tuning parameters, or better avoid local optima. *Hybridization* is a common strategy that combines two or more algorithms into a new, more effective algorithm. The hybridized algorithm often outperforms the individual algorithms used in its creation.^15,16^ A specific application of hybridization was demonstrated by Shi et al,^17^ who combined PSO-quantum and random forest to predict disease progression for patients with idiopathic pulmonary fibrosis using baseline data only.

### PSO

PSO, proposed by Kennedy and Eberhart,^18^ is a prominent nature-inspired metaheuristic algorithm. Despite the introduction of many newer algorithms over the past two decades, PSO remains one of the most widely used optimizers. All nature-inspired metaheuristic algorithms are motivated by natural phenomena or animal behavior. PSO can be visualized as a flock of birds searching for food (the global optimum) on the ground. Each particle (bird) represents a candidate solution for the global optimum and has its own perception of where the food is (local optimum). As the particles explore and exploit the search domain, they share information with each other, guided by two key equations below.

For a swarm of S particles, where 1≤i≤S , let $X_{i}\left(k\right)$ and $V_{i}\left(k\right)$ denote the position and the velocity vectors of the i ^*th*^ particle at the k ^*th*^ iteration, respectively. Define $L_{i}\left(k-1\right)$ as the position vector corresponding to the best objective function value identified by the i ^*th*^ particle up to the (k−1) ^*st*^ iteration, and G(k−1) as the position vector of the best value found by the entire swarm before the k ^*th*^ iteration. At the k ^*th*^ iteration, PSO updates the particle positions and velocities using the following equations

(1) $$X_{i}\left(k\right)=X_{i}\left(k-1\right)+V_{i}\left(k\right),$$

(2) $$\begin{array}{c} V_{i}\left(k\right)={\mathrm{wV}}_{i}\left(k-1\right)\\ \;+c_{1}R_{1}⊗[L_{i}\left(k-1\right)-X_{i}\left(k-1\right)]\\ \;+\;c_{2}R_{2}⊗[G_{i}\left(k-1\right)-X_{i}\left(k-1\right)]. \end{array}$$

Several parameters in equation (2) influence the behavior of PSO. The inertia weight, denoted by w , determines the momentum of particles and their tendency to continue moving in their current direction. Although w can be constant, it is more commonly reduced gradually over iterations, eventually decreasing to zero. The parameters $c_{1}$ and $c_{2}$ are the positive constants that control the influence of the particle’s individual best position, $L_{i}\left(k-1\right)$ , and the global best position, G(k−1) , respectively. It is recommended to set $c_{1}=c_{2}=2$ for effective convergence. $R_{1}$ and $R_{2}$ are the random vectors with components independently drawn from a uniform distribution on [0,1] . The notation⊗ is the element-wise multiplication of two vectors.

Our experience suggests that the number of iterations and the swarm size have a greater impact on the performance of PSO than the choice of the tuning parameters. A larger swarm size allows for broader exploration of the search space, increasing the likelihood of finding a global optimum. In addition, a higher number of iterations give particles more opportunities to refine their search through random perturbations. Users need to specify the swarm size and the duration for which PSO is allowed to run. This duration can be defined by the maximum number of function evaluations, the maximum number of iterations, or the CPU time. The swarm size refers to the number of particles in the swarm that search for the optimum, with each particle representing a candidate solution for the global optimum. In this context, the global optimum is the optimal design with the best design criterion value among all designs for the given setup.

To date, there are only a few of papers that directly apply metaheuristics to design clinical trials. Lange and Schmidli^19^ are probably among the first to use PSO to find optimal designs to estimate parameters in a modified $E_{\max}$ model, along with a standard pharmacokinetic model, to study monoclonal antibodies as they were administered subcutaneously. The second application concerns the celebrated Simon’s two-stage optimal designs for phase II trials,^20^ which have only one set of hypotheses to be tested. Kim and Wong^21^ employed a hybrid version of PSO to better capture the true efficacy of the drug by allowing three sets of postulated alternative hypotheses to be tested in stage 2. The strategy is to find a design that tests only one of the three sets, subject to multiple type I and II errors, and which one will depend on the quality of the results in stage 1. Most recently, Schepps et al.^22^ proposed combining various metaheuristic algorithms to optimize recruitment strategies for global multi-center clinical trials with multiple constraints. Details of these three studies and the optimization problems are in the cited papers, and each is much more complicated than the previous one.

The next section illustrates how metaheuristics can be used to develop improved and more practical designs. We focus on dose-finding designs, a field with a long history, and it is still an active area of research. Wong and Lachenbruch^23^ provide a tutorial on this topic. Many dose-finding designs are often determined numerically without a formal optimality criterion, making it unclear whether the sought design is truly optimal or if the same numerical method would yield the optimal design for another model or criterion. Sometimes, a mathematical approach is used, but this method can be highly sensitive to all aspects of the model assumptions and is difficult to adapt to a slightly altered model. In contrast, metaheuristics address the optimization problem quite independently of the statistical model or design criterion or the nature of the problem.

## New applications of metaheuristics to tackle dose-finding design problems in clinical trials

The aim of a dose-finding trial is to determine a recommended dose for later-phase testing. Researchers increasingly embrace a model-based approach for improved statistical inference over algorithm-based designs, like the 3 + 3 design and its many modifications, that have little statistical justifications.^24^ Our application of metaheuristics focuses on designing phase I/II studies that jointly consider toxicity and efficacy outcomes. The dose–response relationship in these studies is described using nonlinear models, such as the four-parameter continuation-ratio (CR) model.^25,26^ Optimal design problems for the CR model are discussed in works by Fan and Chaloner,^27^ Rabie and Flournoy,^28^ Alam et al,^29^ and Qiu and Wong.^30^ These optimal designs have complex structures, and currently, there is no commercial software to find them.

Let $D=[d_{L},d_{U}]$ denote the interval of study doses, where $0<d_{L}<d_{U}$ are the pre-specified lower and upper limits of the dose range. Suppose the outcome of a patient is trinomial: Z=0 (no efficacy, no toxicity); Z=1 (efficacy without toxicity); and Z=2 (toxicity), and assume that the dose–response relationship $\pi_{j}(d,\theta )=\mathrm{Pr}(Z=j|d,\theta )$ can be plausibly described using a CR model

(3) $$\begin{array}{c} \pi_{0}\left(d,\theta \right)=\left(1-p_{E|T^{c}}\left(d\right)\right)\left(1-p_{T}\left(d\right)\right)\\ \;\;\;\;\;\;\;\;\;\;\;\;=\frac{1}{\left(1+e^{a_{1}+b_{1}d}\right)\left(1+e^{a_{2}+b_{2}d}\right)}, \end{array}$$

(4) $$=\frac{e^{a_{2}+b_{2}d}}{\left(1+e^{a_{1}+b_{1}d}\right)\left(1+e^{a_{2}+b_{2}d}\right)},$$

(5) $$\begin{array}{c} \pi_{2}\left(d,\theta \right)=p_{T}\left(d\right)=\frac{e^{a_{1}+b_{1}d}}{1+e^{a_{1}+b_{1}d}}, \end{array}$$

Equations (3)–(5) are obtained from two logistic regression models, one for the conditional probability of efficacy given no toxicity, $p_{E|T^{c}}\left(d\right)=e^{a_{2}+b_{2}d}/\left(1+e^{a_{2}+b_{2}d}\right)$ , and the other one for the probability of toxicity, $p_{T}\left(d\right)=e^{a_{1}+b_{1}d}/\left(1+e^{a_{1}+b_{1}d}\right)$ .

The CR dose–response relationship is characterized by the parameter vector $\theta ={\left(a_{1},b_{1},a_{2},b_{2}\right)}^{⊤}$ , where $b_{1}>0$ , $b_{2}>0$ , $a_{1}\geq a_{2}$ , and $a_{2}<0$ . Several doses may be of interest to the investigator. The *maximum tolerated dose* ( MTD ) is defined as a 100Γ ^*th*^ percentile of the dose–toxicity curve equation (5), where Γ∈(0,1) is a pre-specified target toxicity level

(6) $$\mathrm{MTD}=\frac{\log\left(\frac{\Gamma }{1-\Gamma }\right)-a_{1}}{b_{1}}.$$

The *optimal biological dose* ( OBD ) can be defined as the dose that maximizes the probability of efficacy without toxicity, that is

(7) $$\mathrm{OBD}=\arg\;\underset{d\in D}{\max}\pi_{1}\left(d,\theta \right).$$

**Figure 1.**
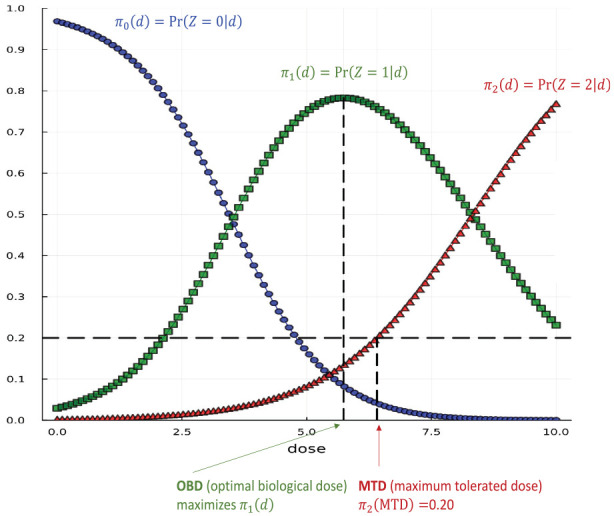
This figure illustrates a dose–response relationship for a four-parameter continuation-ratio (CR) model, determined by ***θ*** = (*a*_1_, *b*_1_, *a*_2_, *b*_2_)^T^, where *b*_1_>0, *b*_2_>0, *a*_1_≥*a*_2_, and *a*_2_<0. The curves in this figure correspond to ***θ*** = (−3.5, 1, −6, 0.72)^*T*^. The dose-response probabilities of the three outcomes are: π_0_(d, ***θ***) (probability of no efficacy and no toxicity, blue curve), π_1_(d, ***θ***) (probability of efficacy without toxicity, green curve), and π_2_(d, ***θ***) (probability of toxicity, red curve). The target toxicity level is set to Γ = 0.2. In this case, MTD = 6.42, and OBD = 5.74.

Figure 1 displays an example of a CR dose–response on the dose interval D=[0,10] and the model parameter vector is known to be $\theta ={\left(-3.5,1,-6,0.72\right)}^{⊤}$ . If Γ=0.2 , a direct calculation shows OBD=5.74 , and MTD=6.42 . In practice, θ is unknown, and we want to design a study that can accurately estimate the quantities of interest, which are OBD and MTD .

Throughout, we consider *continuous* designs as probability measures on D , that is, the designs of the form $\xi =\{\left(d_{i},\rho_{i}\right),\;i=1,\ldots ,K\}$ , where $\rho_{i}\in \left(0,1\right)$ is the allocation proportion for $d_{i}\in D$ with $\sum_{i=1}^{K}\rho_{i}=1$ . Hence, a continuous design is characterized by the number of doses, the specific doses, and the proportions of observations taken at each dose. These proportions are often referred to as weights. The “worth” of a design ξ is evaluated using the Fisher information matrix (FIM)

$$M\left(\xi ,\theta \right)=\sum_{i=1}^{K}\rho_{i}\mu \left(d_{i},\theta \right),$$

where $\mu \left(d_{i},\theta \right)=-E\left[\frac{\partial^{2}\ln\;L\left(\theta ;d_{i}\right)}{\partial \theta \partial \theta^{⊤}}\right]$ is the information for θ from a single patient’s observation at dose $d_{i}$ , and L is the likelihood function based on the assumed statistical model. Importantly, $M^{-1}\left(\xi ,\theta \right)$ is the lower bound for the asymptotic covariance matrix of an efficient estimator of θ .

We consider the following four optimal designs:

I. The unrestricted D -optimal design minimizing −ln|M(ξ,θ)| with dose levels selected from $D=[d_{L},d_{U}]$ .
II. The restricted D -optimal design minimizing −ln|M(ξ,θ)| with dose levels selected from $D_{*}=[d_{L},\mathrm{MTD}]$ .
III. The unrestricted c -optimal design minimizing asymptotic variance of the efficient estimator of OBD , $Var(\hat{OBD})=c^{⊤}\left(\theta \right)M^{-1}\left(\xi ,\theta \right)c\left(\theta \right)$ , where $c\left(\theta \right)=\frac{\partial }{\partial \theta }\mathrm{OBD}\left(\theta \right)$ , with dose levels selected from D .
IV. The restricted c -optimal design minimizing $\mathrm{Var}(\hat{\mathrm{OBD}})$ with dose levels selected from $D_{*}$ .

The CR model is nonlinear, and the design criteria are formulated in terms of FIM, which includes unknown model parameters that need to be estimated. Therefore, the criterion cannot be directly optimized without nominal values for these parameters. Nominal values, which represent best guesses for the model parameters, can be obtained from expert opinions or pilot studies. As a result, the optimal designs for the CR model are *locally* optimal, meaning they depend on the nominal values of θ . To estimate the quantities in equations (6) or (7), we seek a continuous design that minimizes the asymptotic variance of the estimated quantity. This variance is a known convex function of the FIM, which contains the unknown model parameters. A design that minimizes this variance is known as a c -optimal design. Similar to D -optimal designs, there are algorithms for finding c-optimal designs. The complication with the OBD for the CR model is that this dose does not have a closed-form expression. The standard steps for finding the c -optimal design for estimating OBD are as follows: (1) differentiate equation (4) with respect to d and set it equal to zero; (2) solve the equation numerically to obtain the OBD , and (3) invoke the inverse function theorem to obtain the explicit gradient of OBD , which is exactly what is required in the algorithm to find a c -optimal design. However, with metaheuristics, one simply inputs the model, the criterion, and a metaheuristic algorithm, like PSO, automatically finds the desired optimal design.

Table 1 presents PSO-generated locally optimal designs (I–IV) assuming the true value of θ is available (see the dose–response relationships in Figure 1). Specifically, we implemented PSO with the following parameters: (1) the swarm size ( S ) = 25; (2) the maximum number of iterations ( $N_{\max}$ ) = 700; (3) an inertia weight ( w ) varied with iterations, starting at 0.9 and decreasing up to 0.4 if $N_{\max}$ number of iterations were performed: $w_{j}={\left(\frac{N_{\max}-j}{N_{\max}-1}\right)}^{\gamma }$ , where γ=1.5 is a relaxation parameter; (4) cognitive coefficient ( $c_{1}$ )=2.5; and (5) social coefficient ( $c_{2}$ ) = 0.5. The Julia code for generating the dose-finding designs in this article is available on GitHub: https://github.com/yevgenryeznik/CRModel.

**Table 1.** The structure of four locally optimal designs for the dose–response in Figure 1.

Design							
I		II		III		IV	
Dose ( $d_{i}$ )	Weight ( $\rho_{i}$ )	Dose ( $d_{i}$ )	Weight ( $\rho_{i}$ )	Dose ( $d_{i}$ )	Weight ( $\rho_{i}$ )	Dose ( $d_{i}$ )	Weight ( $\rho_{i}$ )
2.22	0.304	2.33	0.409	4.55	0.635	3.61	0.376
5.31	0.449	4.42	0.182	8.33	0.365	6.41	0.624
9.95	0.247	6.41	0.409				

I: unrestricted D -optimal; II: restricted D -optimal; III: unrestricted c -optimal; IV: restricted c -optimal.

**Figure 2.**
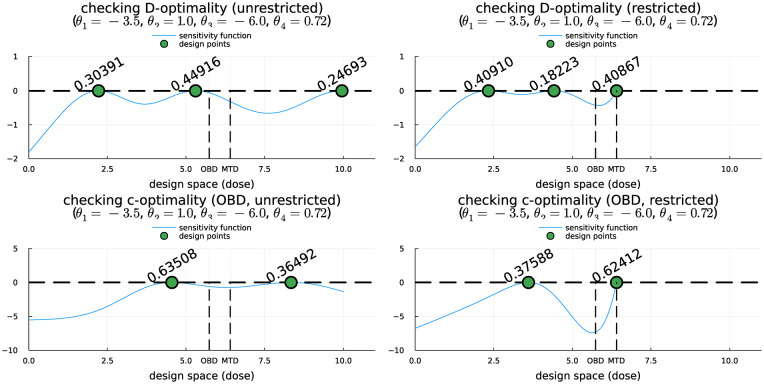
The fulfillment of the GET conditions for the four locally optimal designs.

The optimality of a continuous design found by PSO can then be verified using a technical result known as a General Equivalence Theorem (GET),^31^ which is specific to each convex criterion. To apply this result, we first evaluate the sensitivity function of the design, which is the directional derivative of the convex criterion evaluated at the candidate design in the direction of design with the single dose at d . Each theorem is expressed as an inequality, with the sensitivity function on the left-hand side and 0 on the right-hand side. If the candidate design is optimum with the smallest criterion value among all continuous designs, the GET asserts that the sensitivity function is bounded above by 0 throughout the dose range, with equality at the dose levels of the optimal design.^31^ Figure 2 displays the sensitivity functions of the locally optimal continuous designs found by PSO, confirming their local optimality. For brevity, we omit the derivation of the sensitivity functions for a design for the CR model. Detailed information can be found in design monographs, such as Fedorov,^32^ Pázman,^33^ Berger and Wong,^34^ and Mohanty.^35^

Continuous optimal designs cannot be implemented in practice because they are defined by the percentage of the total number of observations to be taken at specific dose levels. To address this, let n be the pre-determined sample size for the study, and let [x] denote the nearest integer of x . Once an optimal design is selected as $\xi^{*}=\{\left(d_{i}^{*},\rho_{i}^{*}\right),\;i=1,\ldots ,K\}$ , it can be implemented by assigning approximately $\left[n\rho_{i}^{*}\right]$ patients to dose $d_{i}^{*}$ subject to the constraint that $\sum_{i=1}^{K}[n\rho_{i}^{*}]=n$ . As an example, we consider the case when n=40 , and ran a Monte Carlo simulation to compare the statistical properties of four selected locally optimal designs defined by:

- Design $I_{\left(n=40\right)}$ : Unrestricted D -optimal design which randomly assigns 40 patients to doses 2.22, 5.31, and 9.95, in the ratio 12:18:10 (i.e., allocation proportions are 0.30:0.45:0.25 ).
- Design ${\mathrm{II}}_{\left(n=40\right)}$ : Restricted D -optimal design which randomly assigns 40 patients to doses 2.33, 4.42, and 6.41, in the ratio 16:8:16 (i.e., allocation proportions are 0.41:0.18:0.41 ).
- Design ${\mathrm{II}}_{\left(n=40\right)}$ : Unrestricted c -optimal design which randomly assigns 40 patients to doses 4.55 and 8.33, in the ratio 25:15 (i.e., allocation proportions are 0.635:0.365 ).
- Design ${\mathrm{IV}}_{\left(n=40\right)}$ : Restricted c -optimal design which randomly assigns 40 patients to doses 3.61 and 6.41, in the ratio 15:25 (i.e., allocation proportions are 0.38:0.62 ).

To investigate the design operating characteristics, we performed simulations and evaluated the Bias, Standard Deviation (SD), and Root Mean Squared Error ( $\mathrm{RMSE}\;=\;\sqrt{\mathrm{Bia}s^{2}+SD^{2}}$ ) of the estimated MTD and OBD , including the probability of having undetermined maximum likelihood estimates (MLEs) of the CR model parameters. Table 2 summarizes the simulation results. We observe that Design ${\mathrm{IV}}_{\left(n=40\right)}$ is best for estimating MTD ; Design ${\mathrm{III}}_{\left(n=40\right)}$ is best for estimating OBD ; and Design $I_{\left(n=40\right)}$ has the lowest probability of undetermined MLE. Similar results can be obtained for other experimental scenarios to provide useful insights at the study planning stage when investigators are tasked with finding a robust and efficient design for their dose-finding experiment.

**Table 2.** Operating characteristics of the four locally optimal designs with n=40 patients.

	Design			
Characteristic	$I_{\left(n=40\right)}$	$II_{\left(n=40\right)}$	$\mathrm{II}I_{\left(n=40\right)}$	$IV_{\left(n=40\right)}$
MTD —Bias^a^	-0.2414	-0.0516	-0.1754	-0.0709
MTD —SD	0.9711	0.7966	0.8167	0.6723
MTD —RMSE	1.0007	0.7982	0.8353	0.6761
OBD —Bias^a^	-0.0275	0.0535	0.0261	0.1009
OBD —SD	0.7152	0.5892	0.5325	0.5608
OBD —RMSE	0.7158	0.5916	0.5331	0.5698
Pr(NoMLE)	0.002	0.041	0.023	0.043

a Bias = average over 1000 Monte Carlo simulation runs of (point estimate − true value) of the parameter of interest ( MTD or OBD ).

## Conclusion

Dose-finding designs are an active area of research.^36,37^ However, many designs are still determined numerically without a formal optimality criterion. Consequently, it is unclear whether such designs are truly optimal or even generalizable. Sometimes, a mathematical derivation is presented, but this approach can be highly sensitive to model assumptions; if the model changes slightly, the derivation usually cannot be amended.

Metaheuristics address the optimization problem regardless of the statistical model or design criterion and can handle multiple constraints. The dose-finding locally optimal designs reported here are more practical than those reported in the literature for the CR model. For example, Fan and Chaloner^27^ presented optimal designs on an unrestricted dose interval, and Qiu and Wong^30^ found optimal designs that may require doses higher than the MTD . The current PSO-generated designs avoid these issues and protect patients from receiving doses higher than the unknown MTD . If needed, they can also be amended to ensure that the OBD is confined within the therapeutic dose range. Due to the flexibility of the algorithm, PSO can also find other dose-finding designs, such as those discussed in Sverdlov and Wong^38^ for seamless phase I/II clinical trials or trials with a bivariate continuous outcome discussed in Dette et al.^39^ In addition, PSO can be adapted to find designs for randomized multi-arm survival trials with more than one objective,^40^ or to optimally assign subjects to various treatment groups with different response variability.^41^ In the Supplementary Material, we further demonstrate the flexibility of PSO to tackle computationally challenging non–dose-finding design problems.

In conclusion, we hope this article will encourage clinical researchers to learn more about metaheuristics and incorporate them into their research. Metaheuristics have the potential to design more flexible and effective trial designs not only for dose-finding but for any computationally challenging trials, including cluster randomized controlled intervention trials for cancer control,^42^ trial designs for a variance heterogeneity model,^41,43^ modern molecularly targeted early phase oncology trials,^44^ or trial recruitment,^45^ among others. Finally, metaheuristics can also be creatively used to analyze different types of massive complex data^46,47^ and as important tools for machine learning.^8,9^

## Supplemental Material

sj-pdf-1-ctj-10.1177_17407745251346396 – Supplemental material for Nature-inspired metaheuristics for optimizing dose-finding and computationally challenging clinical trial designs Supplemental material, sj-pdf-1-ctj-10.1177_17407745251346396 for Nature-inspired metaheuristics for optimizing dose-finding and computationally challenging clinical trial designs by Weng Kee Wong, Yevgen Ryeznik, Oleksandr Sverdlov, Ping-Yang Chen, Xinying Fang, Ray-Bing Chen, Shouhao Zhou and J Jack Lee in Clinical Trials
