# Supplementary material for: Nature-inspired metaheuristics for optimizing dose-finding and computationally challenging clinical trial designs
Source: Clin Trials. 2025 Jul 12;22(4):422–9. doi: 10.1177/17407745251346396 (PMC12318163; doi:10.1177/17407745251346396)
Supplement: sj-pdf-1-ctj-10.1177_17407745251346396 – Supplemental material for Nature-inspired metaheuristics for optimizing dose-finding and computationally challenging clinical trial designs [file sj-pdf-1-ctj-10.1177_17407745251346396.pdf]

Journal Title
XX(X):1–4

©The Author(s) 2016
Reprints and permission:
sagepub.co.uk/journalsPermissions.nav

DOI: 10.1177/ToBeAssigned
www.sagepub.com/
SAGE

Supplementary Materials: Nature-inspired Metaheuristics for Finding Other Computationally Challenging Designs in Clinical Trials

Weng Kee Wong[1], Yevgen Ryeznik[2], Oleksandr Sverdlov[3], Ping-Yang Chen[4], Xinying Fang[5], Ray-Bing Chen[6], Shouhao Zhou[5], and J. Jack Lee[7]

Applications of PSO to find non dose-finding designs in Clinical Trials

This section shows the utility and flexibility of PSO to tackle other computationally challenging design problems in clinical trials. We demonstrate how PSO can directly extend Simon's 2-stage phase-II designs to 3 or more stages and find more powerful and flexible Bayesian adaptive designs.

Extending Simon's 2-stage phase II designs to multi-stages

Following Simon's work,[1] let n_1 and n_2 be, respectively, the number of patients in the first and second stage in a Simon 2-stage design for a Phase II trial. The total sample size is $N = n_1 + n_2$ and the goal is to find an optimal design, subject to given Type I error (t1e) and Type II error (t2e). The decision rule for rejecting the null hypothesis H_0 that the drug is ineffective and terminating the study is if there are

- r_1 or fewer responses are observed in n_1 patients at the end of first stage, or
- $r = r_1 + r_2$ or fewer responses are observed in N patients at the end of second stage.

Given the error rates, the goal is to determine the optimal values for n_1, r_1, n_2 and r_2 under one of the two objectives in Simon's paper.[1] A direct calculation shows that if the success probability is p, the probability of early termination (PET) after the first stage is

$$PET(p) = B(r_1, p, n_1) = \sum_{x \le r_1} b(x, p, n_1),$$

where $B(\cdot)$ is cumulative binomial distribution and $b(\cdot)$ is binomial probability mass function. Under H_0, the expected sample size of the study is

$$E(N \mid H_0) = n_1 + [1 - PET(p_0)]\, n_2,$$

where p_0 is a specified ineffective response rate, and the probability of rejecting the drug in the study is

$$B(r_1, p, n_1) + \sum_{x=r_1+1}^{\min[n_1, r]} b(x, p, n_1) B(r - x, p, n_2).$$

A greedy search over all possible values of n_1, n_2, r_1 and r_2 was then used to find their optimal values. When there are 3 or more stages, such an approach will be both time consuming and inefficient.

Using PSO to find multi-stage designs for PSO. To apply PSO for solving the problem, we first reformulate the components of the optimization problem as follows:

$$S = (z_1, z_2, \cdots, z_K, \phi_1, \phi_2, \cdots, \phi_K).$$

The first half of the vector represents the cohort size of each stage, satisfying $z_k \in [z_L, z_U]$ and $\sum_{k=1}^{K} z_k \le N_{\max}$, where z_L and z_U are user-defined lower and upper limits for the cohort size of each stage, and $N_{\max}$ is the pre-specified upper limit of the total sample size of the trial. To guarantee z_k's satisfying the summation constraint, we need to check the PSO's particles after updating their positions. Here, we consider projecting the infeasible particles into the feasible region using the following method. At each PSO iteration, if $\sum_{k=1}^{K} z_k > N_{\max}$, then this first half of the vector is transformed by

$$z_k \leftarrow \frac{z_k}{\sum_{i=1}^{K} z_i} \times N_{\max}.$$

The cohort size of each stage is then given by $n_k = [\![z_k]\!]$, where $[\![a]\!] = \lfloor a + 0.5 \rfloor$ denotes the rounding function.

[1] Department of Biostatistics, University of California, Los Angeles, California, USA
[2] Department of Pharmacy, Uppsala University, Uppsala, Sweden
[3] Advanced Quantitative Sciences, Novartis Pharmaceuticals Corporation, New Jersey, USA
[4] Department of Statistics, National Taipei University, New Taipei, Taiwan
[5] Department of Public Health Sciences, Pennsylvania State University, Hershey, Pennsylvania, USA
[6] Institute of Statistics and Data Science, National Tsing Hua University, Hsinchu, Taiwan
[7] Department of Biostatistics, The University of Texas MD Anderson Cancer Center, Houston, Texas, USA.

Corresponding author:
Weng Kee Wong, Department of Biostatistics, University of California, Los Angeles, California, USA.
Email: wkwong@ucla.edu

The latter half of the particle represents the vector of proportions for the cohort size at each stage, satisfying $\phi_k \in [0, 1]$ for $k = 1, \ldots, K$. The cutoff boundary is then calculated as $r_k = [\![\phi_k \cdot n_k]\!]$.

The objective function for finding the optimal K-stage design is

$$\min_S \{E(N|H_0) + M \times \max\{I(\text{t1e} > \alpha), I(\text{t2e} > \beta)\}\} \quad (1)$$

Let the total sample size be $N = \sum_{k=1}^{K} n_k$. The objective function for finding minimax K-stage design is

$$\min_S \left\{ \left[\frac{1}{N} E(N|H_0) + N \right] + M \times \max\{I(\text{t1e} > \alpha), I(\text{t2e} > \beta)\} \right\} \quad (2)$$

where $M >> 0$ is the penalty on those designs not satisfying the requirements of type I error and power. Typically M can be very large, say $M = 10^8$.

Results. We show that PSO can find the same optimal two-stage and three-stage designs, as those obtained from a greedy search in Simon[1] and Chen,[2] respectively. For two-stage optimal and minimax designs, we run PSO with 256 particles for 400 iterations. For finding these three-stage, we used 512 particles and 800 iterations because of the higher-dimensional optimization problems. Other PSO parameters are set to their default values. The numerical results are shown in Table 1 for the case when $(p_0, p_1) = (0.2, 0.4)$ and the error constraints are $(\alpha, \beta) = (0.1, 0.1)$. For this design setup, Simon[1] reported the two-stage optimal and minimax designs are $\{r_1/n_1, (r_1 + r_2)/(n_1 + n_2)\} = \{3/17, 10/37\}$ and $\{3/19, 10/36\}$, respectively, and, Chen[2] reported the three-stage optimal and minimax designs are $\{r_1/n_1, (r_1 + r_2)/(n_1 + n_2), (r_1 + r_2 + r_3)/(n_1 + n_2 + n_3)\} = \{1/10, 6/26, 11/43\}$ and $\{2/16, 5/26, 10/36\}$, respectively.

To assess the efficiency of PSO, we conducted 100 replications for each type of designs and calculated the frequency that PSO successfully found the best designs reported in Simon[1] and Chen.[2] For two- and three-stage minimax designs, PSO was able to find the best design 99 times out of 100 replications. For two-stage optimal designs, the PSO performance was similar but for three-stage optimal designs, PSO was only able to find the optimal three-stage design shown in Chen[2] 39 times out of 100 replications. This may appear discouraging but it is worth noting that (i) the designs found by PSO are all very efficient relative to those in Chen.[2] If we define the EN-efficiency of a design S by $\text{Eff}_{EN}(S) = \frac{E_{S^*}(N|H_0)}{E_S(N|H_0)} \times 100\%$, where S^* is the optimal design,[2] a direct calculation shows 36 of the 61 PSO-generated designs have at least 98.18% EN-efficiency, and the 25 worst PSO-generated designs have approximately 85.73% EN-efficiency, and (ii) these results are generated using the original PSO, the basic version of the PSO algorithm. As is typically expected with metaheuristics, utilizing a variant of PSO can enhance the performance of the original PSO. Our latest research working with modified versions of PSO (i.e., PSO variants) shows that not only can the variants find Simon's designs with three stages faster and more often than the original PSO, but they can also potentially generate Simon's designs with more stages. This is part of our current work and we plan to report more fully with computational details soon.

More powerful generalized Bayesian optimal phase II designs

The previous example demonstrates the use of PSO in expanding the number of stages in the frequentist multi-stage study designs. There are other design methodologies developed for such studies, including those that use the Bayesian paradigm. Bayesian optimal designs incorporate prior and accumulating information as the trial progresses. Thus, Bayesian methods take the "learn as we go" approach, making them innately suitable for clinical trials. In the following example, we illustrate an application of PSO in Bayesian approaches to enhance study flexibility.

A class of well-motivated and flexible Bayesian optimal designs for Phase 2 (BOP2) trials was proposed and studied.[3,4] In assessing the efficacy and toxicity of a drug, interim looks could be added to monitor intermediate results for possible early termination either for toxicity, futility, or efficacy. In contrast to the frequentist approaches that often require strict adherence to pre-specified cohort sizes at each interim stage, Bayesian adaptive designs offer an alternative strategy to accommodate deviations in cohort sizes. Alternative decision boundaries can be calculated based on posterior probabilities for any sample size, allowing for easy adjustments when discrepancies arise between the planned design and actual trial conduct.[5] To meet regulatory needs, recent developments have shifted attention toward ensuring Bayesian designs maintain frequentist type I error rates while maximizing the study power.[3,6] BOP2 designs represent a state-of-the-art methodology and a webpage to interactively find Bayesian Optimal Phase II Design with simple and complex endpoints is freely available at https://biostatistics.mdanderson.org/shinyapps/BOP2/.

The online interactive tool finds Bayesian optimal designs when the efficacy or toxicity measures are continuous, discrete, ordinal and the study is time to event. The user provides the null and alternative hypotheses to be tested, the nominal type I error rate, and a pre-specified vague prior distribution. The user then chooses a simulation setup and the operating characteristics of the design, including the simulated power of the test, are nicely displayed with animation as well. Some drawbacks or limitations are the number of interim looks and the sample size at each stage need to be specified in advance, and the model optimization is performed to find decision boundaries. These restrictions were placed because of the computational burden and lack of a more effective algorithm than the greedy search to simultaneously optimize the number of interim looks and when they occur to optimize the power of the test, or some modifications thereof. Below we show that nature-inspired metaheuristic algorithms can tackle the complicated optimization problem with many more variables in which traditional algorithms cannot.

We used simulation and compared performances of PSO and its variants: PSO-Default,[7] PSO-Quantum,[8] and PSO-Dexp[9] using a single-arm four-stage phase II clinical trial design and compared the original BOP2 approach with

Table 1. Results of finding two- and three-stage optimal and minimax designs. The design requirement is $(p_0, p_1) = (0.2, 0.4)$ and the error constraints are $(\alpha, \beta) = (0.1, 0.1)$

K	PSO Parameters (Swarm, Iterations)	Design Type	PSO Result	T1E	Power	$E(N\|H_0)$	#(achieve)	CPU Time (seconds)
2	(256, 400)	Optimal	3/17, 10/37	0.095	0.903	26.022	99/100	1.68
		Minimax	3/19, 10/36	0.086	0.902	28.263	100/100	1.71
3	(512, 800)	Optimal	1/10, 6/26, 11/43	0.100	0.901	23.860	39/100	32.89
		Minimax	2/16, 5/26, 10/36	0.086	0.901	26.413	99/100	22.88

*T1E: type I error of the design.

*#(achieve): times of the PSO finding the best design over 100 replicates.

*CPU time (seconds): the numerical experiments are conducted in a computer with an Intel Core(TM) i7-13700 2.10 GHz CPU and equipped with 128 GB RAM.

Table 2. Comparison between the original BOP2 design and the extended BOP2 design with PSO algorithms

PSO Parameters (Swarm, Iterations)	Method	λ	γ	PSO Result	T1E	Power	#(achieve)	CPU Time (Seconds)
-	BOP2	0.95	0.621	2/15, 5/25, 8/35, 15/50	0.05	0.881	-	0.6
	PSO-Default	0.936	0.651	2/15, 6/29, 10/39, 14/50	0.05	0.912	193/1000	10.2
(64, 200)	PSO-Quantum	0.931	0.641	2/15, 6/29, 10/39, 14/50	0.05	0.912	10/1000	10.0
	PSO-Dexp	0.938	0.655	2/15, 6/29, 10/39, 14/50	0.05	0.912	62/1000	10.1

*#(achieve): times of the PSO finding the best design over 1000 replicates.

*CPU time (seconds): the numerical experiments are conducted in a high-performance cluster with an Intel(R) Xeon(R) CPU E5-2680 v3 @ 2.50GHz.

the extended BOP2 design. The latter two are variants of PSO; Lukemire et al.[10] demonstrated its usefulness for finding optimal designs for mixed models, and Stehlík et al.[9] proposed a double exponential PSO with informed tuning parameters to find optimal exact designs for a variety of biostatistical problems. The simulations were conducted under a single binary efficacy endpoint, with a nominal type I error rate of $\alpha = 0.05$, and the true response rates under H_0 and H_1 were $p_0 = 0.2$ and $p_1 = 0.4$, respectively. While this example uses a simple binary setting for illustration, PSO can optimize more complex trial designs involving univariate or multivariate outcomes, including discrete and continuous measures of efficacy and toxicity, as well as time-to-event endpoints.

The original BOP2 design requires pre-specifying cohort sizes n_k at each stage. Through grid search optimization, BOP2 identifies the optimal combination of parameters, λ and γ, in the decision criteria of futility stopping

$$Pr(\theta \leq p_0 | D_k) > \lambda \left(\frac{\sum_{i=1}^{k} n_i}{N} \right)^{\gamma}$$

for each stage and maximizes study power while ensuring control over the type I error rate. In this comparison, we utilize a four-stage BOP2 design with cohort sizes of (15, 25, 35, 50). In contrast, in the extended BOP2 design, the cohort sizes n_k will be optimized using PSO; only basic trial design requirements were specified: a cohort size of 15 for the first stage to collect sufficient data for the first assessment, a minimum increment of 8 between stages to avoid too close interim assessments, and a total sample size of 50.

The loss function for PSO algorithms to minimize is defined as:

$$\bar{L}(\lambda, \gamma) = t2e + M \times f(t1e)$$

To maintain control over the type I error rate, a binary step function f is employed. If the type I error rate exceeds the nominal level, $f(t1e)$ will be 1 and the loss function will be penalized and dominated by M. If the type I error rate is controlled, then $f(t1e)$ will be 0. The comparative results, shown in Table 2, indicate that the extended BOP2 design, even with basic requirements, can consistently identify a phase II trial design with enhanced statistical power while maintaining strict control of the nominal type I error rate. Using the same n_1 and N, the extended BOP2 design with PSO algorithms optimizes the interim times and decision boundaries, resulting in an increase in design power from 0.881 to 0.912 (Table 2). Figure 1 illustrates histograms of the power for all designs identified by the three PSO algorithms across 1,000 replications. When rounding the power values to three decimal places, the majority of designs achieve a power of 0.912. The minimum powers of the designs generated by all three PSO algorithms are higher than the power of the original BOP2 design (0.881), underscoring the robustness of the extended approach.

References

1. Simon R. Optimal two-stage designs for phase II clinical trials. *Controlled Clinical Trials* 1989; 10(1): 1–10.
2. Chen T. Optimal three-stage designs for phase II cancer clinical trials. *Statistics in Medicine* 1997; 16(23): 2701–2711.
3. Zhou H, Lee J and Yuan Y. Bop2: Bayesian optimal design for phase II clinical trials with simple and complex endpoints. *Statistics in Medicine* 2017; 36: 3302–3314.
4. Zhou H, Chen C, Sun L et al. Bop2: Bayesian optimal phase II clinical trial design with time-to-event endpoint. *Pharmaceutical Statistics* 2020; 19: 776–786.
5. Lee J and Liu D. A predictive probability design for phase II cancer clinical trials. *Clinical Trials* 2008; 5(2): 93–106. DOI: 10.1177/1740774508089279.
6. Lin R and Lee J. Novel bayesian adaptive designs and their applications in cancer clinical trials. In Bekker A, Chen DG and Ferreira J (eds.) *Computational and Methodological Statistics and Biostatistics: Contemporary*

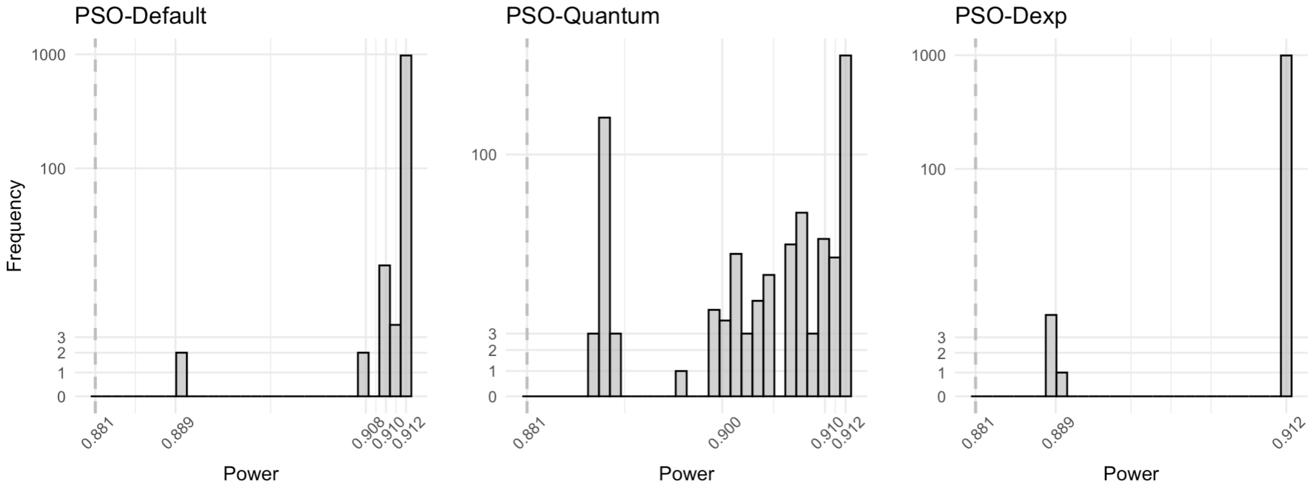

Figure 1. Power of all designs identified by the extended BOP2 design across the 1000 replications (Table 2). The dashed vertical line is the power of the design identified by the original BOP2 design. The powers achieved by the extended BOP2 design are higher than the power of the BOP2 design.

Essays in Advancement. Springer International Publishing, 2020. pp. 395–426.

7. Kennedy J and Eberhart R. Particle swarm optimization. In *Proceedings of ICNN'95 - International Conference on Neural Networks*. pp. 1942–1948. DOI:10.1109/ICNN.1995.488968.
8. Sun J, Xu W and Feng B. A global search strategy of quantum-behaved particle swarm optimization. *IEEE Conference on Cybernetics and Intelligent Systems* 2004; : 111–116DOI:10.1109/ICCIS.2004.1460396.
9. Stehlík M, Chen PY, Wong WK et al. A double exponential particle swarm optimization with non-uniform variates as stochastic tuning and guaranteed convergence to a global optimum with sample applications to finding optimal exact designs in biostatistics. *Applied Soft Computing* 2024; 163: 111913.
10. Lukemire J, Mandal A and Wong W. d-QPSO: A quantum-behaved particle swarm technique for finding d-optimal designs with discrete and continuous factors and a binary response. *Technometrics* 2019; 61(1): 77–87.
